# Treatment of Pathological Bone Fractures in a Patient with McCune-Albright Syndrome 

**DOI:** 10.1155/2013/589872

**Published:** 2013-11-28

**Authors:** Jana Kollerova, Tomas Koller, Zuzana Zelinkova, Ludmila Kostalova, Juraj Payer

**Affiliations:** ^1^5th Department of Internal Medicine, Faculty of Medicine, Comenius University, Ruzinovska 6, 82606 Bratislava, Slovakia; ^2^2nd Department of Pediatrics, Faculty of Medicine, Comenius University, Limbova 1, 83340 Bratislava, Slovakia

## Abstract

McCune-Albright syndrome is a rare genetic disorder with typical skeletal and endocrine manifestations. The disease course is complicated by recurrent fractures resulting from polyostotic fibrous dysplasia and the treatment is thus primarily directed at the reduction of the risk of fractures. However, due to the complex mechanism of the skeletal damage the standard antiporotic therapeutics are ineffective. We report here a case of a 31-year-old female, diagnosed with the McCune-Albright syndrome in early childhood. She was suffering from extensive bone involvement, complicated by recurrent fractures despite the treatment with bisphosphonates. In addition, the disease course was complicated by the impairment of several endocrine functions—precocious puberty, hyperestrogenism, and hyperthyroidism for which a total thyroidectomy was performed. During the operation, two enlarged parathyroid glands were removed. This resulted in severe hypocalcaemia in the postoperative period with a need for supplementation with very high calcium and vitamin D doses. After this episode, the patient has remained free of fractures. We discuss here the corrected thyroid function, the supplementation with unconventionally high doses of vitamin D and calcium, and the termination of bisphosphonates treatment as presumable factors contributing to the reduced fracture risk in this patient.

## 1. Introduction

McCune-Albright syndrome belongs to rare genetic diseases. It results from sporadic early-onset postzygotic somatic mutations in the GNAS1 gene and is characterized by the triad of bone dysplasia, skin hyperpigmentation, and various endocrine organs involvement. The extent and degree of involvement of affected tissues are heterogeneous due to the mosaicism of the genetic mutations and therefore the phenotypes can differ in each individual patient. In the past years, important insights have been gained into the pathogenetic mechanisms of the disease [1]. However, the therapeutic decision-making remains complicated and no clear standards exist [2]. Patients with McCune-Albright syndrome reach the adult age with a significant burden of the disease that continuously reduces their quality of life. Skeletal deformities, fractures, hyperthyroidism, and hyperestrogenism are just few of the many challenges in the management of these patients. Here, we present our experience with the management of these particular complications in an adult female patient with McCune-Albright syndrome.

## 2. Case Report

31-year-old female was diagnosed with McCune-Albright syndrome in early childhood and her disease course was complicated by hyperestrogenism, fibrous bone dysplasia, slight skin alterations with hyperpigmentations “café au lait” on lips and on the back, thyroid hyperfunction, and cardiac manifestations with sinus tachycardia and long QT interval.

The detailed description of the clinical presentation and initial treatment of this patient has been reported previously [3, 4]. Briefly, the disease manifested itself as peripheral precocious puberty at the age of 6 months resulting from hyperestrogenism. Precocious puberty treatment by medroxyprogesterone acetate helped to ensure her proper sexual development but she still needs exogenous gestagens treatment to counterbalance the estrogen overproduction from the right ovarian cysts.

**Figure 1:**
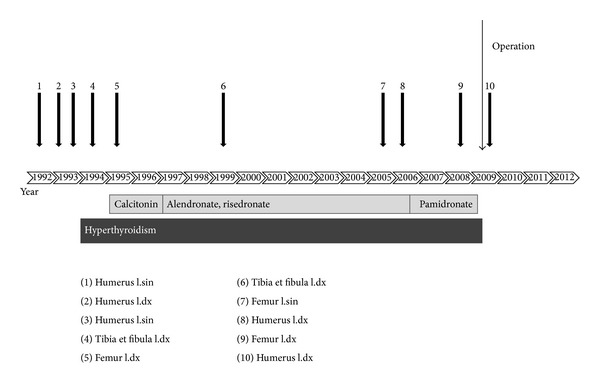
Timeline of the fractures and the relevant treatment from birth till present. Numbers 1 to 10 represent each one of the fractures; all of them occurred in affected parts of long bones. (1) Humerus l.sin, (2) humerus l.dx, (3) humerus l.sin, (4) tibia et fibula l.dx, (5) femur l.dx, (6) tibia et fibula l.dx, (7) femur l.sin, (8) humerus l.dx, (9) femur l.dx, and (10) humerus l.dx.

Cystic bone alterations were first detected at the age of 5 years. Identification of bone lesions together with the present endocrine and skin changes led to the diagnosis of McCune-Albright syndrome. At the age of 6 years, first signs of body dissymmetry were observed, mainly on the skull. Subsequently, long bone lesions complicated the course of the disease with cysts growth and expanding bone alterations. Within the period of 4 years, from the age of 11 to 14, the patient suffered from recurrent long bone fractures. In order to prevent further fractures, calcitonin treatment was initiated at the age of 14. Two years later, calcitonin was replaced by bisphosphonates, alendronate, and later risedronate, combined with calcium and vitamin D. This resulted in improvement of bone pain and the followup showed no fractures up to the age of 23. At the age of 24 the patient suffered a pathological infraction of left femur and a fracture of right humerus. During the following 5 years, she had several events of pathological fractures of long bones (Figure 1). All fractures occurred in bone affected by high bone turnover, as documented by the scintigraphy (Figure 3) which showed the extension of the bone involvement virtually over the entire skeleton. At this point, the oral bisphosphonates were changed for intravenous pamidronate but no positive effect on the fractures rate was observed. Vitamin D and calcium were both substituted during all that time. Bone turnover markers remained elevated despite the treatment, probably as a result of a high bone turnover in polyostotic fibrous dysplasia (Figures 3 and 4). Slightly elevated level of parathormone was assigned to the vitamin D deficiency, with improvement after substitution. Levels of calcium and phosphorus fell into the reference range.

**Figure 2:**
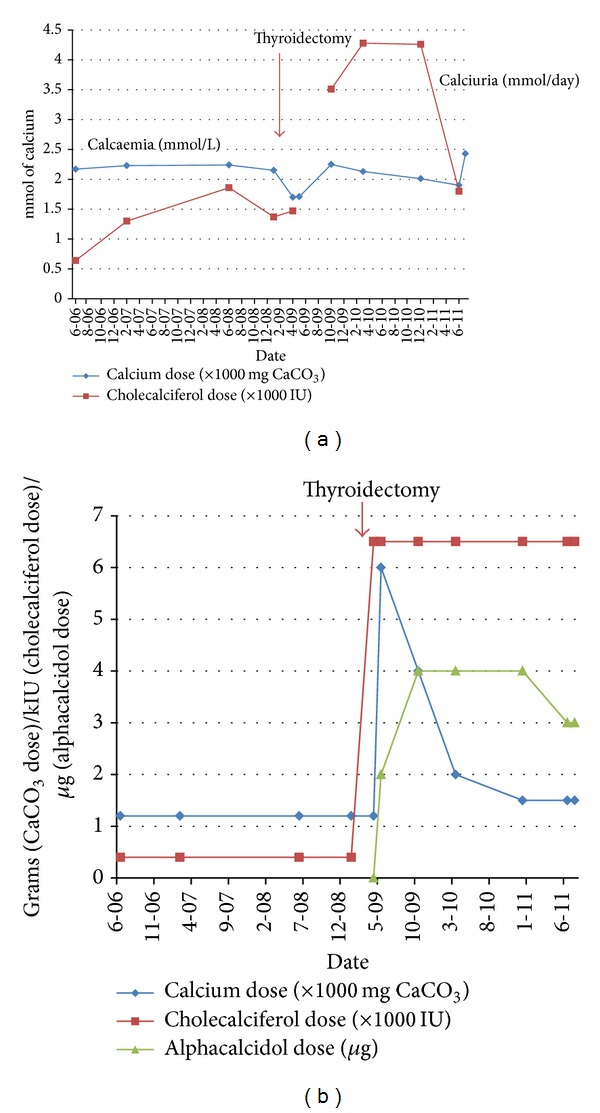
Calcaemia and calciuria in the perioperative period (a). Supplementation with calcium and vitamin D in this period (b). *y* axis shows a time line with specified datums of the measurements in the year of thyroidectomy (2009).

**Figure 3:**
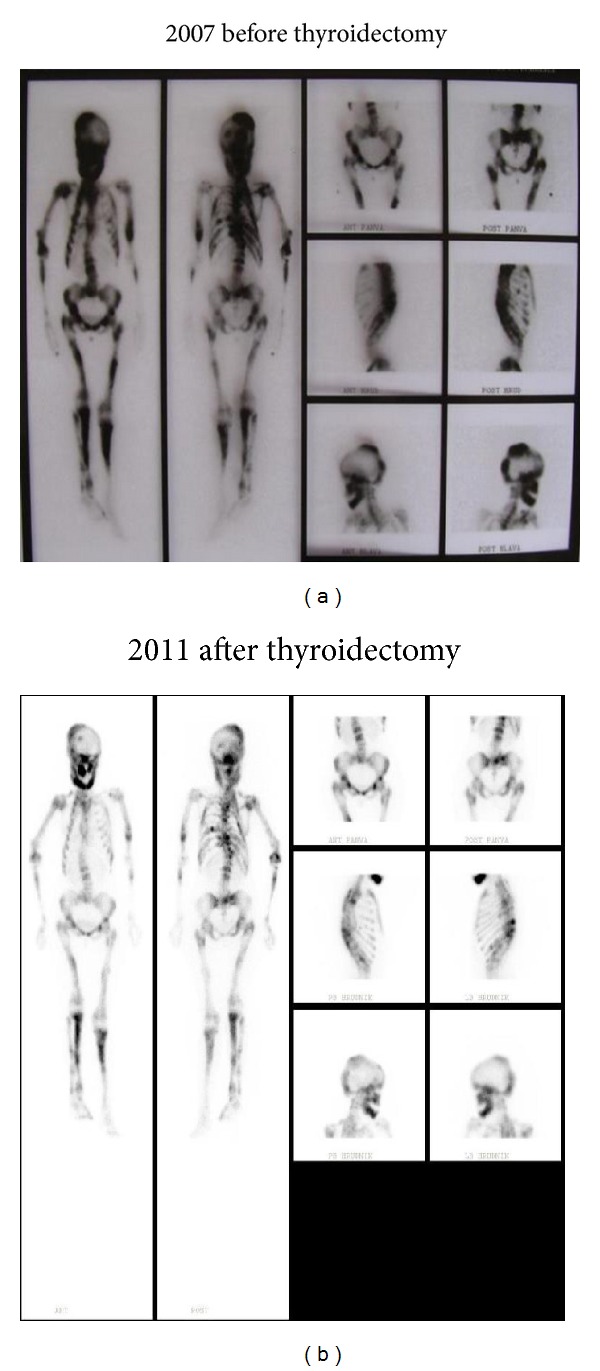
Skeletal scintigraphy with 99mTcHTD before and two years after thyroidectomy.

**Figure 4:**
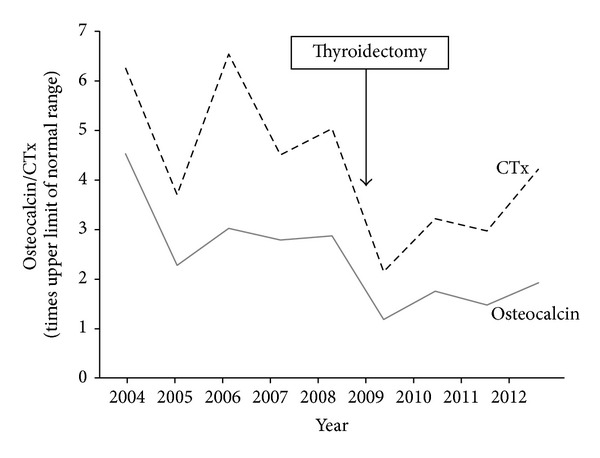
Changes of markers of bone turnover after thyroidectomy. Full line: osteocalcin; dotted line: serum collagen type 1 cross-linked C-telopeptide (CTx).

A third complication of the disease course, hyperthyroidism, was diagnosed at the age of 13 years. The patient had a toxic multinodular goiter due to the hyperfunctioning nodules. In order to reach euthyroidism, antithyroid medication with carbimazole and later thiamazole was used. Despite this treatment, the fluctuating function with unpredictable flares of hyperthyroidism was present. The lack of control of the thyroid function and the concerns about the malignant transformation of the multinodular goiter have finally led to the decision to perform a thyroidectomy that was performed in April 2009. The histological examination of the removed thyroid gland showed thyroid nodular hyperplasia without dysplastic or malign signs. During the operation, two markedly enlarged parathyroid glands were removed because of suspicion for adenomas. This was not confirmed by the histological examination which showed nodular thyroid hyperplasia and small parathyroid glands without histological abnormalities. In the postoperative period, the patient developed severe hypocalcaemia with tetany. At this point, the concentration of parathormone was low but within the reference range. The dose of calcium and vitamin D needed to preserve patient asymptomatic was very high (Figure 2). Additionally, patient was treated for hypothyroidism and hyperestrogenism and bisphosphonates treatment was discontinued. In the early postoperative period, patient suffered from one pathological fracture of right humerus, but since then no fractures were observed.

## 3. Discussion

We report here an unexpected reduction of fractures rate in a patient with McCune-Albright syndrome, who after thyroidectomy and parathyroidectomy received a supplementation with unconventionally high doses of calcium and vitamin D.

Polyostotic fibrous dysplasia is characterised by multiple patchy areas of bone lysis and sclerosis. Consequently, the disease course is complicated by multiple skeletal fractures and deformities with limited success of different therapeutic procedures [2]. In the present case, calcitonin and later bisphosphonates temporarily helped to decrease the fractures rate at the age of 14, but the fractures of long bones appeared again at the age of 24 and the pharmacological interventions were ineffective in the reduction of the risk of fractures. Currently, the patient is free of fractures for an unusually long period of time after she underwent the thyroidectomy and parathyroidectomy followed by the supplementation with high doses of vitamin D and calcium. We discuss here some factors that altogether have had contributed to the fractures risk reduction in this particular patient.

First factor that presumably contributed to the reduction of fractures rate in this patient is the achievement of euthyroidism after a long period of fluctuating thyroid function. Chronic thyroid hormone excess can cause clinically significant bone mineral loss through direct stimulation of bone resorption. In addition, it contributes to bone formation by birth of multicellular units, harbouring the osteoclast resorption and osteoblast formation sequence [5]. Hyperthyroidism can be associated with hypercalciuria and less often hypercalcaemia [6]. Our patient had a long lasting history of a difficult-to-control hyperthyroidism which was accompanied by a state of high bone turnover. This high bone turnover may be related to the polyostotic fibrous dysplasia itself but the fact that it decreased after thyroidectomy suggests a connection of the high bone turnover with hyperthyroidism. In addition, the documented episode of hungry bone in the postoperative period might be explained by a reduction in bone resorption preceding the bone formation that ensued from the corrected thyroid function. Thus, the high thyroid activity could have worsened the bone condition through ongoing high bone turnover state.

Second, the hypoparathyroidism resulting from the resection of parathyroid glands together with the supplementation with high doses of vitamin D and calcium postoperatively could have had an additional positive effect on the bone turnover in this patient. Typically, the osteomalacia associated with McCune-Albright syndrome is hypophosphatemic and hyperphosphaturic, but this was not the case of our patient. The primary hyperparathyroidism is also unlikely, considering the laboratory findings of normocalcaemia, hypocalciuria, hypophosphaturia, and slight hypovitaminosis D. We ascribe these findings to a secondary hyperparathyroidism with fluctuating parathormone levels depending on the level of patient's compliance in the use of the medication. As discussed here above, the patient's high need for calcium, phosphorus, and vitamin D in the postoperative period was likely caused by the so-called “hungry bone syndrome.” Thus, the saturation of calcium and vitamin D and the decreased parathormone levels might have contributed to the final decrease of the fractures rate.

Third, the long-term bisphosphonates treatment itself can increase the risk of atypical fractures [7–9] which represents another putative risk factor of increased fractures rate that needs to be taken into consideration in our patient. Evaluation of criteria for atypical fractures is difficult in a patient with fibrous bone dysplasia as the bone structure and X-ray appearance are disrupted by the disease. Although all the fractures that our patient experienced during the long-term bisphosphonates treatment were in the affected bone, there are some arguments against a causal relationship of the cessation of the bisphosphonates treatment and the reduction of fractures rate. First, the thyroidectomy was followed by suppression and not an increase of bone turnover. Second, the effect of bisphosphonates on the bone formation persists for several years after the cessation of treatment and such a rapid effect of the treatment discontinuation is unlikely. Therefore, it does not seem likely that the cessation of bisphosphonates played a major role in the mechanism by which the fractures risk was reduced in this patient.

The other factor that might have had impact on the bone metabolism in this complicated patient is hyperestrogenism. Hyperestrogenism has been shown to have negative impact on osteoresorption, and in the McCune-Albright syndrome, the bone lesions show the presence of estrogen and progesterone receptors in osteogenic cells [10]. Whether estrogen receptors are unique to McCune-Albright syndrome or, alternatively, a property of any activated, dedifferentiated, or neoplastic bone cells is unclear. Considering that, in our patient, the hyperestrogenism was present for long time and was not modified by any recent intervention, it is difficult to ascribe this particular factor an important role in the fractures risk reduction.

In conclusion, management of bone dysplasia in patients with McCune-Albright syndrome represents a clinical challenge. Based on our experience, we suggest attempting to correct all possible contributing factors to bone dysplasia, that is, thyroid function together with adequate supplementation of calcium and vitamin D. The question remains whether the long-term bisphosphonates treatment is beneficial for these patients and what is the best timing of their discontinuation.
